# A Three-Step Diagnostic Algorithm for Alopecia: Pattern Analysis in Trichoscopy

**DOI:** 10.3390/jcm14041195

**Published:** 2025-02-12

**Authors:** Alexander C. Katoulis, Georgia Pappa, Dimitrios Sgouros, Effie Markou, Antonios Kanelleas, Evangelia Bozi, Demetrios Ioannides, Lidia Rudnicka

**Affiliations:** 1 2nd Department of Dermatology and Venereology, Medical School, National and Kapodistrian University of Athens, “Attikon” General University Hospital, 124 62 Athens, Greece; gpappa100@gmail.com (G.P.); disgo79@gmail.com (D.S.); effie.markou@hotmail.com (E.M.); antkanel@med.uoa.gr (A.K.); bozi.derma@gmail.com (E.B.); 2 1st Department of Dermatology and Venereology, Medical School, Aristotle University, 541 24 Thessaloniki, Greece; dem@auth.gr; 3 Department of Dermatology, Medical University of Warsaw, 02-091 Warsaw, Poland; lidiarudnicka@gmail.com

**Keywords:** alopecia, diagnosis, algorithm, trichoscopy, hair disorders, clinical practice

## Abstract

**Background/Objectives**: Alopecia is a common and distressing hair loss condition that poses a major diagnostic challenge. While histopathology is the gold standard, its invasive nature limits its routine use. Trichoscopy, a non-invasive imaging technique, has shown promises in diagnosing and differentiating the various alopecia subtypes. However, existing diagnostic algorithms primarily rely on dermoscopic findings. To address this, we developed a novel, three-step algorithm that integrates clinical and trichoscopic features and employs pattern analysis as a diagnostic tool. **Methods**: A comprehensive literature review was conducted to identify key trichoscopic features associated with different alopecia types. The gathered data were used as a base for the description of trichoscopic features and patterns for each subtype of alopecia, either scarring or non-scarring. **Results**: The proposed algorithm is analyzed into three steps. In the first step, alopecia is categorized by distribution into: patchy, patterned, or diffuse. In the second step, it distinguishes between scarring and non-scarring alopecia based on the absence or presence of follicular ostia, respectively. Lastly, in the third step, alopecias are distinguished based on specific trichoscopic clues, allowing for the identification of distinct trichoscopic patterns. **Conclusions**: The three-step diagnostic algorithm for alopecia, utilizing clinical and dermoscopic findings, performs a pattern analysis in trichoscopy, leading to a dermoscopic diagnosis with great confidence, and minimizing the need for invasive diagnostic procedures.

## 1. Introduction

Dermoscopy is a non-invasive, in vivo diagnostic method that utilizes a light source combined with a magnifying lens (×10–20). It allows for the identification of colors, patterns, and morphological structures that are not visible to the naked eye, providing a detailed visualization of the epidermis, the dermoepidermal junction, and the papillary dermis [1]. Trichoscopy is the implementation of dermoscopy specifically on the scalp, focusing on the detailed examination of hair and scalp disorders. The utilization of trichoscopy as a diagnostic tool for hair disorders has emerged as a significant application of dermoscopy, second in importance only to dermato-oncology. Alopecia, a common, multifaceted condition with varying etiologies, is not only a medical challenge but also a source of profound psychosocial distress for affected individuals [2]. Traditionally, diagnosis relied heavily on a combination of patient history, physical examination, trichogram analysis, and histologic examination [3]. Histopathology is considered the gold standard for diagnosis, being also the basis for the current classification of scarring alopecia into neutrophilic, lymphocytic, or mixed [3]. Nevertheless, skin biopsy is a costly, invasive procedure that is restricted by the selection of the appropriate site. These constraints make it impractical as a diagnostic tool in everyday clinical practice. Recent advances in trichoscopy, however, have provided a new and improved method for the diagnosis of alopecia. By enabling detailed visualization of the scalp and hair shafts, trichoscopy allows for a bridge between the clinical and histopathological characteristics of various forms of alopecia. This non-invasive technique offers significant advantages, including improved diagnostic accuracy, the ability to track disease progression over time, and enhanced patient follow-up [4]. As a result, the need for biopsy is minimized and restricted only for diagnostic verification in difficult or ambiguous cases. Building upon this knowledge, we aimed to develop a new algorithm to simplify the process and improve diagnostic accuracy.

## 2. Materials and Methods

We conducted a meticulous literature review, using established scientific databases such as PubMed and Scopus to design a clinically relevant, and practical three-step diagnostic algorithm for alopecia. The search terms and keywords were carefully designed by pairing each specific type of alopecia, combined with phrases such as “diagnosis”, “trichoscopy”, “dermoscopy”, “dermoscopic features”, or “trichoscopic features”. We also manually reviewed studies reference lists from key articles to identify additional studies that might not have been retrieved during the initial database search. The search was designed to retrieve articles that detailed trichoscopic features associated with various subtypes of alopecia, as well as studies proposing diagnostic frameworks. Furthermore, we examined existing diagnostic algorithms to identify gaps, limitations, and opportunities for improvement. By critically evaluating both trichoscopic data and algorithmic approaches, we ensured that our proposed diagnostic tool is both innovative and grounded in evidence-based practice.

**Figure 1:**
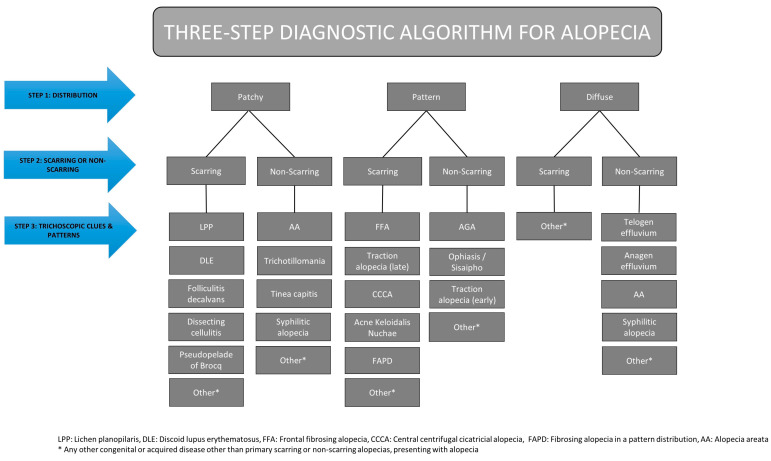
Schematic representation of the three-step diagnostic algorithm for alopecia.

It is important to note that our study primarily focused on primary alopecias, both scarring and non-scarring. As such, secondary alopecias caused by inflammatory or systemic diseases (e.g., systemic lupus erythematosus, sarcoidosis, bullous pemphigoid), trauma, physical factors (e.g., burns or radiation), as well as congenital and acquired pediatric alopecias (e.g., aplasia cutis congenita, loose anagen hair syndrome, triangular alopecia), and scalp nevi or neoplasms were not individually analyzed. Instead, these conditions have been collectively categorized as “other” in our schematic representation (Figure 1). By narrowing our scope to primary alopecias, we aimed to develop a more targeted diagnostic tool.

## 3. Results

The formulated algorithm integrates both clinical manifestations and trichoscopic findings to achieve a more comprehensive and streamlined diagnostic approach. It consists of three steps, which are analyzed below:

**Step 1: What is the hair loss distribution?**

The initial step involves classifying the hair loss based on its distribution. Patchy hair loss typically presents as discrete, often circular or oval-shaped areas of hair loss. It is seen in conditions such as alopecia areata (AA); trichotillomania; tinea capitis; discoid lupus erythematosus (DLE); lichen planopilaris (LPP); folliculitis decalvans; dissecting cellulitis; pseudopelade of Brocq; traction alopecia; and syphilitic alopecia, which is distinguished by its characteristic “moth-eaten” borders [5,6,7,8]. Pattern hair loss follows a predefined location pattern, such as the involvement of the frontotemporal area and the vertex in male pattern hair loss, parietal regions, and the vertex in female pattern hair loss [9]. Frontal fibrosing alopecia (FFA) presents with a band-like recession of the frontotemporal or other marginal hairline [10]. Acne keloidalis nuchae typically affects the occipital scalp [11], while central centrifugal cicatricial alopecia (CCCA) starts on the vertex or crown and progressively spreads outward in a centrifugal pattern [12]. Fibrosing alopecia in a pattern distribution (FAPD) presents with hair loss in a centroparietal distribution (bitemporal) or affects the crown and vertex, often sparing the frontal hairline [13]. Ophiasis is characterized by a band-like hair loss in the occipital scalp extending bilaterally to the temporal areas [14]. Conversely, in sisaipho, which represents reverse ophiasis, hair loss affects most of the scalp but spares the occipital and temporal regions [14]. Lastly, diffuse alopecia involves a more uniform, diffuse thinning of hair across the entire scalp, as is commonly seen in both telogen and anagen effluvium [15,16], as well as in diffuse alopecia areata and alopecia areata incognita [17]. Less commonly, it can also occur in syphilitic alopecia [8].

**Step 2: Is it scarring or non-scarring alopecia?**

In the second step, the clinician must determine whether the alopecia is scarring or non-scarring, based on both clinical and trichoscopic findings; a distinction which is crucial as it influences both the diagnosis and the treatment approach. Scarring alopecia is identified clinically by white, shiny, cicatricial skin, and trichoscopically by the absence of follicular ostia [18]. In contrast, non-scarring alopecia presents with normal-appearing skin, and evenly distributed follicular openings both clinically and dermoscopically [18].

**Step 3**: **What are the specific trichoscopic clues and the trichoscopic pattern?**

The third and final step capitalizes on the unique trichoscopic clues that have been described for each specific alopecia subtype. In primary scarring alopecias, trichoscopic diagnostic clues vary. In LPP, the dominant features are areas of violaceous perifollicular erythema, commonly associated with perifollicular scale or casts (adherent to the proximal end of the hair shaft, and attached to the scalp), due to follicular hyperkeratosis; while in the chronic stages, one can observe milky-red areas, corresponding to advanced fibrosis and related vascular changes [19]. FFA differs dermoscopically from LPP in that there is mild perifollicular reddish erythema and scaling; presence of lonely hairs, which represent remaining, isolated terminal hairs in hairless areas, reflective of advanced follicular loss; absence of vellus hair (in contrast to traction alopecia); and presence of red dots in the eyebrow region, indicative of vascular changes associated with early, reversible follicular involvement [20,21,22,23,24]. In contrast to the other primary lymphocytic scarring counterparts, DLE exhibits distinct trichoscopic features, including follicular plugs—thickened, keratin-filled follicular openings. Red dots, indicative of early, reversible hair follicle involvement, correspond to dilated infundibula containing keratin plugs or emerging hair shafts, surrounded by dilated vessels and red blood cell extravasation on histopathology. Additional characteristic findings include perifollicular post-inflammatory hyperpigmentation, appearing in a honeycomb or speckled brown pattern, and blue-gray pigmentation, reflecting melanin incontinence [24,25,26,27,28,29,30]. Regarding primary neutrophilic scarring alopecias, folliculitis decalvans showcases unique trichoscopic features like hair tufts, where multiple hairs (5–6 or more) emerge from a single opening in the cicatricial scalp; moderate to severe perifollicular scaling, or more often, hair casts, surrounding the proximal end of the hair shaft; pustules at the periphery of the lesion; and yellow crusts, likewise predominantly at the periphery, which reflect dried exudates from the pustules [18,27,28]. Interestingly, both in clinical practice and in the literature, a distinct folliculitis decalvans–LPP phenotypic spectrum has been recognized [29]. Clinicians must be aware of this entity, as it represents a cicatricial alopecia with overlapping trichoscopic features of both conditions. It is characterized by the successive or simultaneous presence of neutrophilic-associated findings, such as pustules, greasy crusts, and follicular tufts, alongside lymphocytic-associated features, including perifollicular erythema and perifollicular scaling [29]. Dissecting cellulitis manifests with follicular pustules, yellow crusts, keratotic plugs, and featureless yellow areas on erythematous, boggy hairless plaques [28,31]. Acne keloidalis nuchae is characterized by pustules or pseudofolliculitis, and ingrown hairs located in the occipital area of the scalp [32]. Traction alopecia in its late, scarring stages exhibits hair casts -white tubular structures composed of keratin and cellular debris- that encircle the hair shaft and can move freely along its length. Additionally, vellus hairs are preserved, as they cannot be retracted and therefore remain unaffected [33]. CCCA reveals perifollicular gray/white halos, corresponding to the concentric fibrosis around the affected follicles, and pinpoint white dots, correlating to the acrosyringeal and follicular openings [34]. FAPD exhibits the features previously described for LPP, however resulting in hair loss following a male or female pattern distribution [13]. On the other hand, pseudopelade of Brocq lacks distinct trichoscopic features, apart from white cicatricial skin and the absence of follicular openings, thus being a diagnosis of exclusion [35].

Non-scarring alopecias also present with specific trichoscopic clues that vary by subtype. In alopecia areata, distinctive trichoscopic features are: black dots, which represent broken hairs at the level of the scalp, with the upper portion of the hair root remaining anchored to the follicular opening, giving the macroscopic appearance of a macrocomedo; “exclamation mark” hairs, which are also broken hairs that have a broader distal end and a narrower, tapered proximal end, corresponding to the acute arrest of the anagen phase; both black dots and exclamation mark hairs are markers of acute, rapidly progressive disease; yellow dots, which correspond to accumulations of keratin and sebum within dilated follicular ostia, may be seen during the subacute and chronic phase of the disease; coudability hairs, which represent long hairs with proximal hair shaft tapering, also result from anagen phase arrest; and thin, non-pigmented hairs signifying the initiation of the hair regrowth phase [36,37,38]. Androgenetic alopecia exhibits: a characteristic hair shaft thickness heterogeneity of more than 20%, which represents the coexistence of thick terminal hairs and thin vellus hairs due to the process of follicular miniaturization; an increased number (>20%) of hair follicles with single terminal hairs, suggestive of reduced number of terminal hairs per follicular unit; increased vellus hairs; and perifollicular pigmentation, presenting as brownish halo surrounding the hair follicles, likely due to perifollicular micro-inflammation. The aforementioned findings can be observed, although with differing distribution patterns, in either male or female pattern hair loss [38]. Traction alopecia in its early, non-scarring stages, can be identified trichoscopically by the presence of hair casts, which result from mechanical stress that causes the detachment of hyperkeratotic perifollicular rings from the scalp; and broken hairs of varying lengths, reflecting ongoing physical trauma and breakage caused by repetitive tension on the hair [39,40]. Telogen effluvium, which is often triggered by systemic stressors such as pregnancy, severe acute or chronic disease, or nutritional deficiencies, is characterized by excessive shedding of telogen hair, due to increased proportion (20–50%) of follicular transition to the telogen phase. It clinically presents with a diffuse thinning of hairs over the entire scalp with a normal (<20%) variability in hair thickness on trichoscopy, a feature that differentiates it from AGA, where significant hair shaft thickness heterogeneity is typically observed [18,38,41]. Often, short regrowing hairs may be present [41]. There are no specific dermoscopic findings and it is also a diagnosis of exclusion. Anagen effluvium, hair loss occurring after the acute arrest of anagen phase, due to cancer chemotherapy, radiation, and toxic chemicals can be identified by black dots, representing cadaverized anagen hair, and different types of dystrophic or broken hairs, indicating damage to the rapidly dividing hair matrix [16]. Among these are coiled hairs, which reflect structural abnormalities caused by the weakening of the hair shaft; pigtail hairs, characterized as shorter, tightly coiled hairs; and tapered hairs, which demonstrate progressive thinning of the shaft, originating at the point of anagen arrest and extending toward the site of breakage [16,42]. Trichotillomania, a compulsive hair-pulling disorder, manifests trichoscopically with broken hair at different lengths, reflecting the inconsistent timing of hair breakage; black dots representing remnants of broken hairs; and “flame hairs” with frayed distal ends, caused by the pulling force applied to the hair shafts [41,43]. Other forms of damaged hair commonly seen in trichotillomania include tulip hairs, with their broader distal ends resembling a tulip; hair powder, consisting of fine hair remnants or debris; and the V-sign, where two hairs emerge from a single follicular opening [43]. Tinea capitis, a fungal scalp infection, is characterized by black dots (broken hair resulting from hair weakening caused by endothrix fungal invasion); scales; comma and zigzag hairs, which display bent, or irregular wavy patterns, respectively, due to structural weakening of the hair shaft caused by fungal damage; Morse code hairs exhibit interrupted transverse lines along the hair shaft, corresponding to areas of fungal invasion; and corkscrew hairs, which are spiral-shaped and indicative of hair shaft distortion [44]. Lastly, syphilitic alopecia, a manifestation of secondary syphilis, exhibits non-specific trichoscopic features that overlap with those of other alopecias [8]. These include zigzag and pigtail hairs, broken hairs, black dots, empty hair follicles, and short regrowing hairs. Given this diagnostic overlap, syphilitic alopecia—much like syphilis itself—is often regarded as a “great imitator” [8].

**Figure 2:**
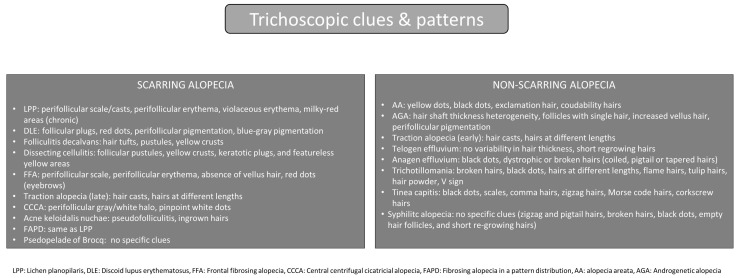
Trichoscopic clues and patterns by type, for scarring and non-scarring alopecia.

In order to act as the basis for pattern analysis, trichoscopic patterns for scarring and non-scarring alopecias have been developed. Each trichoscopic pattern consists of 3 components, namely distribution of hair loss, presence or absence of follicular openings, and trichoscopic clues. The data for each of the three components is obtained during the respective stages of the three-step process. The three-step diagnostic algorithm is summarized in Figure 1 and Figure 2.

Examples of the application of the three-step algorithm are provided below.

**Figure 3:**
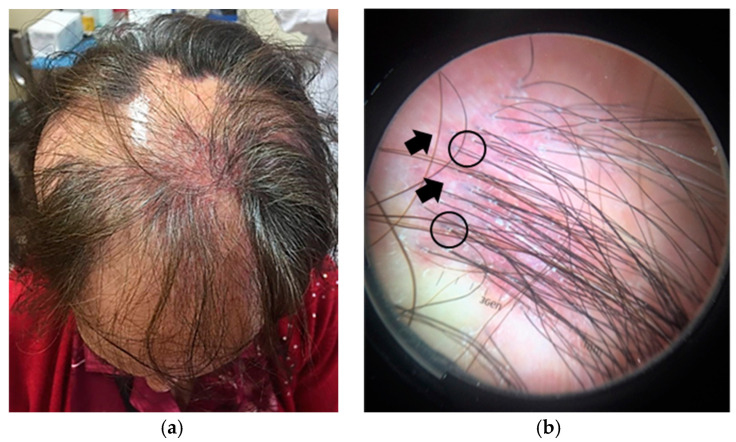
(**a**) Confluent patches of scarring alopecia with foci of violet erythema in a 60-year-old woman. (**b**) Trichoscopy revealing violet erythema (black arrows) and perifollicular scaling (black circles).

Example 1. A 60-year-old female patient with confluent patches of alopecia on the frontoparietal scalp (Figure 3a).

Clinical examination shows a patchy alopecia, with white, shiny, cicatricial scalp skin. Trichoscopic evaluation reveals the absence of follicular openings, indicating that it is a scarring alopecia. Specific trichoscopic features include foci of violet erythema and perifollicular scaling (Figure 3b). The combination of these findings leads to a clinico-dermoscopic diagnosis of a patchy, scarring alopecia, namely LPP.

**Figure 4:**
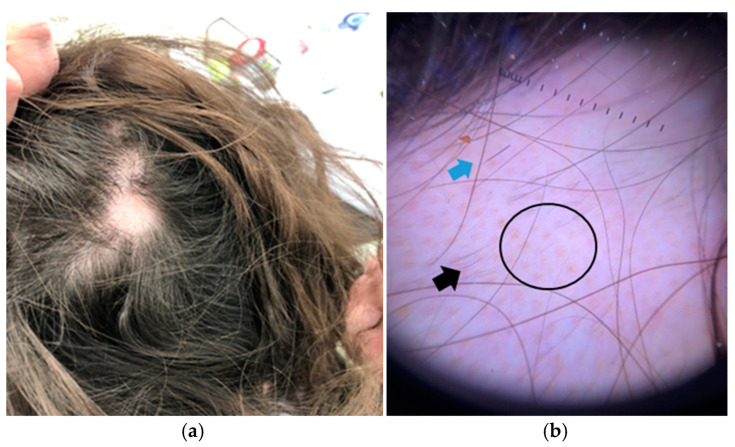
(**a**) Single alopecic plaque on the vertex of a 37-year-old woman. (**b**) Trichoscopy revealing yellow dots (black circle), exclamation mark hairs (blue arrow), and short re-grown hairs (black arrow).

Example 2. A 37-year-old woman presenting with a circular alopecic plaque on the vertex of the scalp (Figure 4a). Clinical examination shows a patchy alopecia, with normal-appearing skin.

Trichoscopic evaluation reveals the presence of evenly distributed follicular openings, suggestive of a non-scarring alopecia. Specific trichoscopic features include yellow dots, exclamation mark hairs, and short vellus, re-grown hairs (Figure 4b). The combination of these findings leads to a clinico-dermoscopic diagnosis of a patchy, non-scarring alopecia, namely AA.

**Figure 5:**
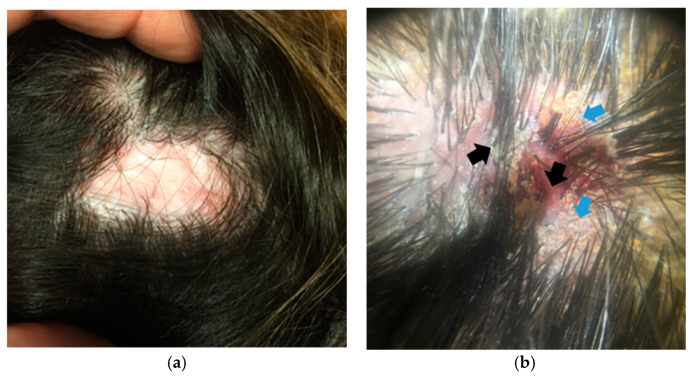
(**a**) A single plaque of scarring alopecia in a 36-year-old woman. (**b**) Trichoscopy revealing hair tufts (black arrows) and yellow greasy scales (blue arrows) at the periphery of the alopecic plaque.

Example 3. A 36-year-old woman presenting with patchy alopecia on the vertex of the scalp (Figure 5a). Clinical examination shows a patchy alopecia, with white, shiny, cicatricial scalp skin.

Trichoscopic evaluation reveals the absence of follicular openings, indicating that it is a scarring alopecia. Specific trichoscopic features from the periphery of the plaque include hair tufts, yellow, greasy crusts, and erythematous erosions (Figure 5b). The combination of these findings leads to a clinico-dermoscopic diagnosis of a patchy, scarring alopecia, namely folliculitis decalvans.

**Figure 6:**
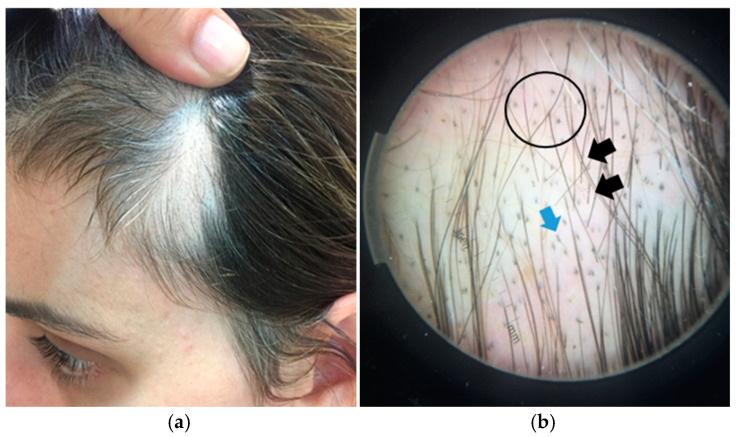
(**a**) Single alopecic plaque on the temporal area of an 18-year-old woman. (**b**) Trichoscopy revealing black dots (black circle), hairs at different lengths (black arrows), and flame hairs (blue arrows).

Example 4. An 18-year-old woman presenting with an ovoid hairless plaque on the left temporal area of the scalp (Figure 6a). Clinical examination shows patchy alopecia, with normal-appearing skin.

Trichoscopic evaluation reveals the presence of evenly distributed follicular openings, indicating that it is a non-scarring alopecia. Specific trichoscopic features include black dots, hairs at different lengths, and a variety of broken hairs, including flame hairs (Figure 6b). The combination of these findings leads to a clinico-dermoscopic diagnosis of patchy, non-scarring alopecia, namely trichotillomania.

## 4. Discussion

Through our literature review, it becomes evident that although numerous studies have proposed dermoscopic clues for each type of alopecia [18,19,20,21,22,23,24,25,26,27,28,29,30,31,32,33,34,35,36,37,38,39,40,41,42,43,44], only a limited number of diagnostic algorithms exist, predominantly based on the analysis of dermoscopic features [45,46]. Although these algorithms serve as foundational tools in clinical practice, they have several limitations that our new algorithm aims to address.

Inui proposed a two-step algorithm for the diagnosis of alopecia [45]. The first step involves determining the presence or absence of follicular ostia, which helps differentiate between scarring and non-scarring alopecia [45]. In the second step, specific dermoscopic findings are identified, and based on them, possible dermoscopic diagnoses are provided for either scarring or non-scarring alopecia [45]. This approach, while useful, does not incorporate clinical information regarding the distribution of hair loss and it is based on the recognition of trichoscopic features rather than trichoscopic patterns.

Rudnicka and Rakowska described the 3-A system for the differential diagnosis of hair loss [46]. This includes a clinical differentiation into diffuse, focal cicatricial, or non-cicatricial alopecia in the first step, and a differentiation based on dermoscopic findings in the second step [46]. While this system provides a structured framework, it is often considered too complex, time-consuming, and requiring memorization.

Our three-step diagnostic approach offers a comprehensive and rational framework for alopecia diagnosis and enhances diagnostic accuracy and flexibility. Most importantly, our algorithm describes trichoscopic patterns for scarring and non-scarring alopecia, enabling the application of pattern analysis in the trichoscopy of alopecia. Although histopathology remains the diagnostic gold standard, trichoscopy is not only a crucial diagnostic tool but also correlates well with histopathological findings and, in certain cases, can provide a definitive diagnosis. A notable example is the differentiation between LPP and DLE, both of which exhibit a dense perifollicular lymphocytic infiltrate and share subtle histopathologic differences [47]. Trichoscopy allows for a clear distinction by highlighting the characteristic trichoscopic patterns unique to each condition [19,20,21,22,23,24,25,26,27,28,29]. Unlike previous algorithms [45,46], our proposed algorithm emphasizes a holistic approach that merges traditional trichoscopic features with broader clinical observations, ensuring that no key diagnostic clues are overlooked. This integration is particularly beneficial in cases where trichoscopic findings are ambiguous, or patients present with overlapping features of different types of alopecia. This approach builds upon existing methodologies by providing a more adaptable decision-making process, allowing for greater adaptability to individual patient presentations.

However, there are several limitations that must be addressed. A major limitation is the absence of histopathological validation. Another significant challenge is the reliance on dermoscopic expertise. The algorithm assumes that clinicians are proficient in identifying and interpreting trichoscopic patterns, which may vary widely based on their training and experience. Lastly, in order to establish its generalizability, further evaluation across diverse demographic groups and clinical settings is required.

To validate its effectiveness, we propose testing the algorithm in controlled clinical environments with dermatologists of varying expertise, from trainees to experienced practitioners. By comparing diagnostic accuracy and consistency before and after applying the algorithm, its impact on clinical decision-making can be objectively measured. These findings would not only support its clinical utility but also advocate for its inclusion in standardized diagnostic pathways and dermatology training programs. Integrating this algorithm into medical education could significantly enhance learning outcomes, equipping trainees with a structured, evidence-based approach to diagnosing alopecia. This would empower future dermatologists to handle complex cases with greater confidence and precision.

Furthermore, the algorithm’s design presents an opportunity for integration with artificial intelligence (AI). Machine learning models trained on trichoscopic images and diagnostic patterns outlined by the algorithm could facilitate the development of an AI-assisted diagnostic tool. Such a tool would provide clinicians with reliable, instant second opinions, particularly in complex or ambiguous cases while reducing diagnostic variability and errors. The adoption of AI-powered trichoscopy could be of utter importance, particularly in under-resourced areas or practices without access to dermatology specialists; more specifically, it could significantly reduce the time to diagnosis and improve patient outcomes in underserved populations, ensuring wider availability of high-quality care.

## 5. Conclusions

In conclusion, our proposed three-step diagnostic algorithm offers a structured and evidence-based approach to addressing the complexities of alopecia diagnosis. It enables clinicians to systematically classify hair loss based on both clinical and trichoscopic findings, leading to a diagnosis promptly and reliably. Its implementation in everyday clinical practice can improve patient evaluation, and enhance diagnostic precision, ultimately leading to more effective therapeutic interventions and patient outcomes. Integrating this diagnostic tool into the medical education and training curriculum for dermatology residents could equip future dermatologists with practical skills to address the diagnostic challenges posed by various forms of hair loss. This, in turn, would raise the standards of patient care.

## Data Availability

The data presented in this study are available on request from the corresponding author due to the sensitive nature of the information derived from patient data.
